# DAILY – A personalized circadian *Zeitgeber* therapy as an adjunctive treatment for alcohol use disorder patients: results of a pilot trial

**DOI:** 10.3389/fpsyt.2025.1477895

**Published:** 2025-02-07

**Authors:** Naomi Springer, Lisa Echtler, Paul Volkmann, Anisja Hühne-Landgraf, Jasmin Hochenbleicher, Eva Hoch, Gabi Koller, Dominic Landgraf

**Affiliations:** ^1^ Circadian Biology Group, Molecular Neurobiology, Department of Psychiatry and Psychotherapy, LMU University Hospital, LMU Munich, Munich, Germany; ^2^ Munich Medical Research School, LMU Munich, Munich, Germany; ^3^ Centre for Neural Circuits and Behaviour, University of Oxford, Oxford, United Kingdom; ^4^ Cannabinoid Research and Treatment Group, Department of Psychiatry and Psychotherapy, LMU University Hospital, and Division of Clinical Psychology and Psychological Treatment, Department of Psychology, LMU Munich., Munich, Germany; ^5^ IFT Center for Mental Health & Addiction Research, Munich, Germany; ^6^ Department of Psychiatry and Psychotherapy, LMU University Hospital, LMU Munich, Munich, Germany

**Keywords:** circadian, chronotherapy, alcohol use disorder (AUD), addiction, relapse, depression, personalized therapeutic approach

## Abstract

**Background:**

Disturbances of circadian rhythms and everyday structures are hallmarks of alcohol use disorder (AUD). Recurring activities such as sleep, activity, and meals represent time cues, so called *Zeitgebers*, that synchronize the circadian system. With our newly developed therapy approach for AUD patients, we aim to reduce relapses and alcohol consumption in AUD patients undergoing a withdrawal program by strengthening their circadian rhythms.

**Methods/Design:**

We aim to achieve the improvement of circadian rhythms with personalized structure plans for daily *Zeitgeber* activities, which are used in parallel with the standard therapy at our clinic. The DAILY pilot study is a six-week monocentric trial with a controlled, randomized, single-blinded, parallel-group design. 41 AUD patients participated in the pilot study and were randomly assigned to an intervention group (IG) or a control group (CG).

**Results:**

The variability of daily activities was significantly lower in the IG group than in the CG group. Of the IG participants, two had relapsed during the study (<12%), whereas ten relapsed in the CG group (>60%). The relapsing IG participants recorded a total of three alcohol consumption days, while the CG participants recorded 52 consumption days. Abstinent participants showed significantly less variability in the times of their daily activities than relapsed participants. In addition, our analyses indicate that craving for alcohol is related to variability in the time of getting up and that craving and temporal variability increase before relapses. Moreover, the general well-being of the IG participants improved more than that of the CG participants.

**Discussion:**

The data from this pilot study show that following a personalized daily structure plan helps AUD patients to remain abstinent during withdrawal therapy. The analyses indicate that temporal variability of daily activities and the risk of relapse are correlated. On the one hand, adherence to the daily structure may prevent relapse, on the other hand, an increase in variability may be a helpful predictor of approaching relapse. In our view, these data justify a continuation of the DAILY study with the addition of further measurements such as the determination of endogenous circadian rhythms.

**Clinical trial registration:**

https://drks.de, identifier DRKS00019093.

## Introduction

Harmful use of alcohol and alcohol use disorder (AUD) lead to increased morbidity and mortality. According to the World Health Organization (WHO), 5.1% of all disorders, diseases, injuries, and 5.3% of all deaths are attributable to alcohol consumption (1). In recent years, evidence emerged that there is a bidirectional relationship between circadian rhythms and alcohol consumption (2). On the one hand, disrupted circadian rhythms are considered a strong risk factor for the development of AUD. For example, disturbances of circadian sleep-wake cycles, as frequently observed in subjects with a late chronotype and shift workers, have been linked to increased prevalence of harmful use of alcohol and AUD (3). Moreover, polymorphisms in the clock genes *CLOCK, PER1, PER2*, and *PER3* genes have been associated with problematic alcohol consumption (4–7) and *PER1* shows significantly different expression levels in GABAergic inhibitory medium-sized spiny neurons of the *Nucleus accumbens* of AUD patients (8). On the other hand, individuals with AUD almost always suffer from disorders of the circadian system, such as disturbed sleep times, impaired sleep quality, altered molecular rhythms, and altered daily rhythms of neuroendocrinological functions in different regions of the brain (9). Importantly, circadian rhythms are also affected during alcohol withdrawal (10, 11) and alcohol-dependent patients have a higher risk of relapse during withdrawal if their internal clock is disturbed (9).

In humans, the master circadian clock is located in the suprachiasmatic nucleus (SCN) in the anterior part of the hypothalamus (12–14). The SCN acts like a conductor of an orchestra and sets all other cellular clocks in the rest of the body to an appropriate period and phase (15). This also affects the daily rhythms of brain regions that regulate reward and whose dysfunction is involved in the development of addiction. Poor circadian synchronization between brain regions can impair their communication (16, 17), which can lead to behavioral deficits and possibly substance dependence.

Synchronization of endogenous circadian rhythms with environmental 24-hour cycles is achieved through so called *Zeitgebers* (German: time givers) (18). The strongest stimuli that serve as *Zeitgebers* are light and food, whereby light acts directly on the SCN. In a simplified model, the SCN, in turn, determines when hunger arises, and the ingested food then entrains peripheral clocks. However, endogenous circadian rhythms can only synchronize stably and permanently with the environment and with each other if the *Zeitgebers* occur at consistent times each day. Irregular time signals result in recurring shifts in the endogenous rhythms, which lead to permanent desynchronization and thus to limited functionality of physiological and neuronal processes. Based on these relations, we designed the therapy study DAILY (**D**epression **A**lcohol **Il**lness Therap**y**) (19).

DAILY aims to identify personal circadian characteristics of AUD patients in order to design individualized daily structure plans regarding meal and bedtimes that AUD patients are encouraged to adhere to during their withdrawal therapy, especially in the transition phase between clinic and home or outpatient therapy. As our therapy study concentrates on the positive psychological effects of structured routines plus the values of a strengthened circadian system, we assume that subjects in our study benefit in two ways from the therapy: firstly, from increased self-efficacy expectations and reduced subjective stress, and secondly, from the reduction of physical conditions that promote and maintain alcohol consumption behavior. We further believe that the strengthening of circadian rhythms has not only positive effects on alcohol craving, but also on often accompanying symptoms of AUD which further facilitate alcohol consumption, such as depressive mood and sleep quality. The specific primary working hypotheses for the pilot study are:

1. The DAILY intervention leads to a reduction in the temporal variability of daily activities as well as to a reduction in alcohol craving, alcohol consumption and relapse.
2. Since elevated temporal variability of daily activities increases alcohol craving and relapse risk, both effects of the intervention are interrelated.
  We also examined the following secondary hypotheses:
3. The DAILY intervention leads to an improvement in accompanying symptoms of AUD including impaired sleep quality, increased depressive symptoms, reduced self-efficacy expectations, and poor blood-values that are associated with harmful alcohol consumption.
4. The DAILY intervention helps participants maintain compliance and remain in the treatment study.
5. The DAILY intervention reduces the temporal variability of daily activities without affecting the individual chronotype.

## Materials and methods

The study was conducted according to the previously published protocol (19). Brief summaries and possible differences to the previously described protocol as well as a detailed description of the statistical analyses applied are given below.

### Participants

The participants were recruited according to the previously defined eligibility and exclusion criteria (19). In brief, participants had to have an ICD-10 F10.2 (alcohol dependence) diagnosis, be between 15-75 years of age and, in the case of other dependence disorders (except nicotine), be free from use of these other substances for at least 12 months. A diagnosis of MDD (excluding psychosis and suicidal ideation) was not a prerequisite but was suitable for inclusion. Exclusion criteria included inability to consent to the study, pregnancy, shift work, medical reasons for inability to independently comply with the intervention, blindness, psychiatric diagnoses other than AUD and MDD, use of benzodiazepines, agomelatine, or medically prescribed cannabinoids, and failure to meet inclusion criteria.

### Recruitment

Recruitment was carried out as described previously (19). Briefly, potential participants were recruited both at the specialist ward for addiction disorders and the outpatient clinic for substance use disorder of the Clinic for Psychiatry and Psychotherapy at the University Hospital of Munich.

### Randomization

Participants who were eligible to participate in the study were assigned to either the intervention group (IG) or a sham control group (CG) based on a computer-generated randomization list according to rules described previously (19).

### Study Design

The study design corresponds to that which has already been published (19).

#### Baseline assessment

After signing the informed consent, regardless of group affiliation, an assessment of the baseline status was carried out with all included participants (questionnaires and blood sampling). Subsequently, participants of the IG and CG underwent different protocols of the same scope.

#### DAILY intervention

During the next appointment, subjects of the intervention group were educated about the circadian clock and its relevance for the body as well as its role in AUD. At the end of this appointment, participants received a blank day structure diary to fill out for one week.

Thereafter, the diary data was evaluated in a further personal meeting together with the participants and a daily structure plan was drawn up with regard to eating, bed, and sleeping times based on identified time clusters in which certain daily activities occurred particularly frequently and possible restrictions (e.g., due to working hours or other obligations). In the following four to five weeks, participants were asked to adhere to their personalized plans as accurately as possible and to continue to keep the day structure diary. As some participants were discharged from the ward or the outpatient clinic over the course of the study, it was in some cases necessary to adjust their day structure plan. To ensure compliance and encounter upcoming questions, each participant was contacted via telephone or in person on a weekly basis. This represents a difference to the previously published study protocol, in which only one telephone contact was assumed after half the time (19).

#### Control intervention

Participants of the control group joined appointments to discuss the role of advertisement of legal substances in AUD and other addictions. They were also encouraged to complete the daily structure diary. However, there was no discussion about the data and no instructions were given on how to adhere to a specific structure. As in the IG, the participants in the CG were also contacted every 1-2 weeks to maintain compliance and to clarify any questions.

#### Final appointment

At the end of the study, a last individual appointment was arranged for participants of both groups. As not all participants were still on site at our clinic, it was not possible to make the final appointment with each participant after an exact number of study days. Participants who had a later appointment than planned were asked to continue the intervention until the appointment and to continue collecting all diary data. Therefore, in contrast to the previously published protocol (19), in some cases the study lasted up to seven weeks instead of six weeks. All diaries that had not been submitted yet were collected. Additionally, the questionnaires from the baseline assessment were repeated, and another blood sample was taken. If participants were in long-term therapy institutions further outside of Munich, this last appointment was conducted via telephone and questionnaires and diaries as well as the results of blood samples taken externally were transferred by fax, mail, or email.

### Outcome measures

#### Day structure diary

The diary provides a tabular basis to track the timing of daily recurring activities such as the times of eating, going to bed, falling asleep, waking up, and getting up (19). Additionally, participants had the opportunity to report sleep quality from the previous night. The last 22 participants included (CG: 11, IG: 11) were also asked about their highest subjective craving for alcohol on each day.

In addition to the paper form of the diary described in the previous protocol, an additional and optional form of communication, the messenger service ‘Wire’ (Wire Swiss GmbH, Zug, Switzerland), was introduced during the course of the study to ensure seamless and real-time data collection. A pseudonymized account was set up for all participants who wished to use the app which was then used for 1:1 communication with the study staff. It was also possible to send reminders regarding actualization of the data if participants chose this option. ‘Wire’ was implemented by the end of the trial, so that only four participants worked with this form of communication.

#### Questionnaires

Questionnaires were used to determine alcohol consumption behavior (AUDIT, EuropASI, TLFB), depressive mood (HAMD, IDS-SR), self-efficacy (SWE), sleep quality (PSQI) and chronotype (MCTQ) (19).

#### Blood values

Blood samples were collected to determine the De Ritis ratio (Glutamic Oxaloacetic Transaminase (GOT)/Glutamate-Pyruvate-Transaminase (GPT)), Gamma GT (GGT), Carbohydrate-Deficient Transferrin (CDT), and Mean Corpuscular Volume (MCV). Contrary to the published protocol, Alkaline Phosphatase (aP), Hemoglobin (Hb), and Vitamin B12 were not assessed.

### Statistics

Not all participants provided all data, which is why there were variations in the sample size for individual analyses.

Differences were considered significant when p < 0.05.

The Kruskal-Wallis test with Dunn’s multiple comparison test with preselected comparisons of CG completers vs. CG drop-outs and IG completers vs. IG drop-outs was used to test whether there were baseline differences between the completing and withdrawing subjects within the CG and the IG. This non-parametric test was chosen due to the very different sample sizes of the respective groups. The results are shown in **Supplementary Table 1.**

**Figure 1:**
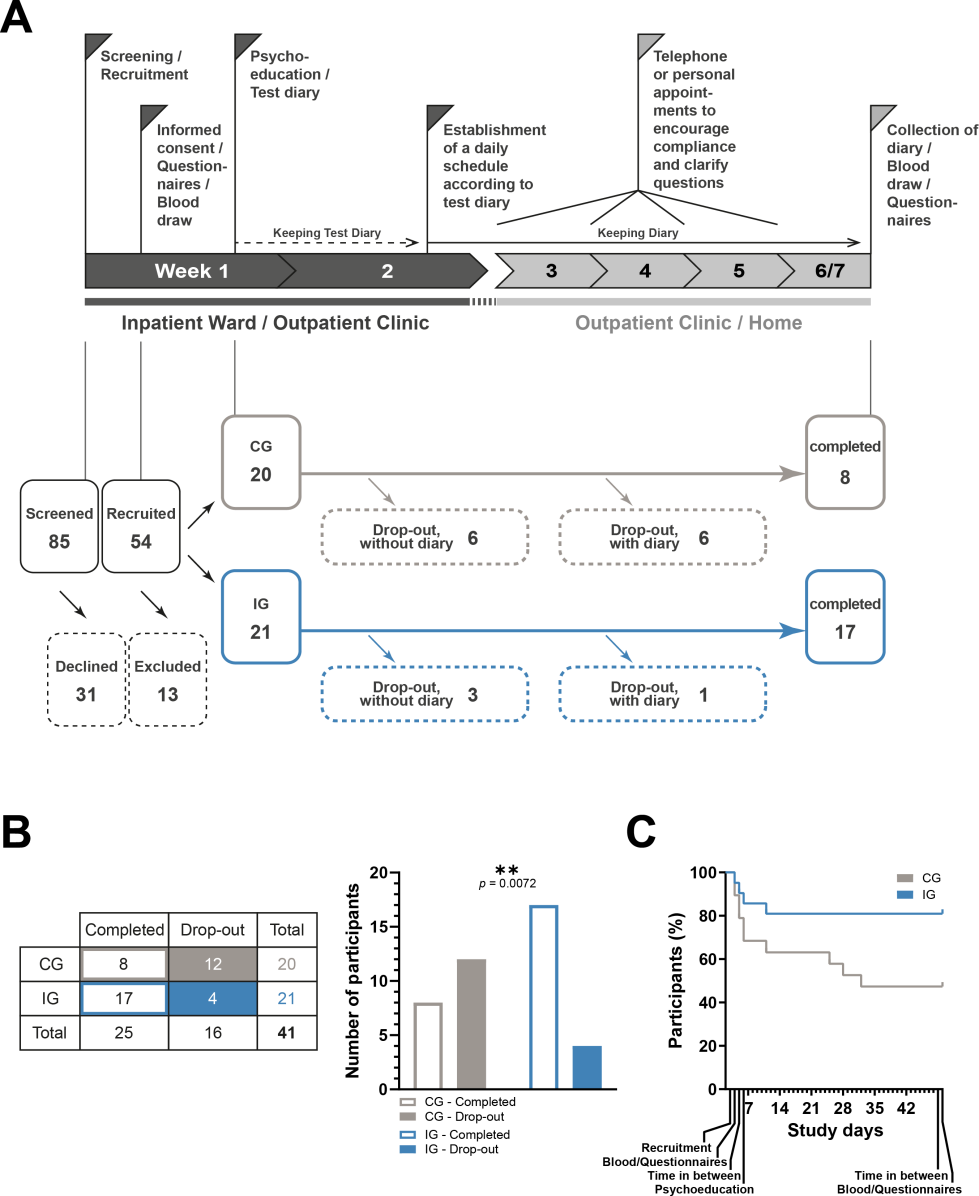
Study timeline and cohort diagram. **(A)** Top: Study timeline as published in (19) with modification. In short, screening, recruitment, the first blood sample, and the first interview with questionnaires take place in week 1. Psychoeducation is then carried out and the participants are given blank diaries to record their daily activities. These are filled out over the course of week 2 and then an individualized daily structure plan is drawn up based on the entries, which the IG participants should adhere to in the subsequent course of the study. During weeks 3-6, all participants are asked to continue filling in the diaries and receive weekly or biweekly reminders in person or via telephone. Afterwards, a final appointment is arranged within one week after finalization of the diaries, so that the end of the study is reached after 42-48 days. At this appointment, another blood sample is taken, and the questionnaires are completed. Bottom: 85 potential participants were screened, 31 of whom declined to take part in the study (category 5). The remaining 54 were recruited for the study, 13 of whom were later found to meet exclusion criteria (category 4). The remaining 41 participants were divided into 20 controls and 21 intervention participants. Of the 20 controls, 12 participants dropped out, 6 of whom did not submit diaries, 6 of whom submitted diaries, and 8 completed the study. Of the 21 intervention participants, 4 participants dropped out, 3 of whom did not submit diaries, 1 of whom submitted diaries, and 17 completed the study. **(B)** Left: Table showing the number of control and intervention subjects who completed the study or dropped out. Right: The ratio between completers and drop-outs is significantly different in the control group and the intervention group. Chi-square test, **<0.01. **(C)** Percentage decrease in study participants in both groups over the course of the study period.

**Figure 2:**
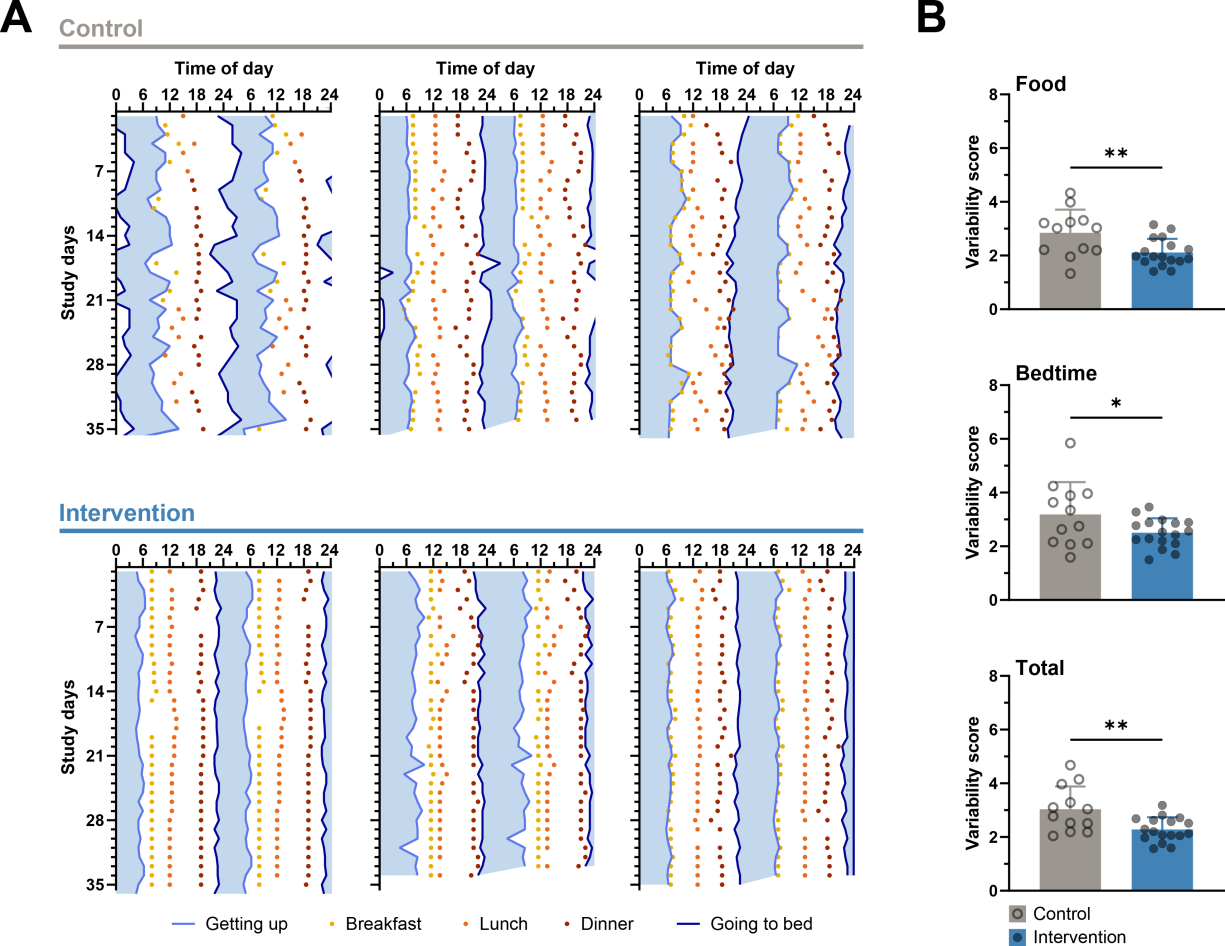
Lower variability in the times of daily activities in the intervention group. **(A)** Representative double-plotted actograms with diary data on sleeping and eating times of three control subjects (top) and three intervention subjects (bottom). Plotted on the abscissa are the self-reported times of going to bed and getting up with the time presumably spent in bed in between (blue shading) as well as the self-reported times of breakfast, lunch, and dinner. The ordinate shows the study days from study week 2, in which the diaries were started to be kept. Participants in the interventions group show lower day-to-day variability in meals and bedtimes than participants in the control group. **(B)** The variability scores of meals, bedtimes, and both together are significantly lower in the intervention group than in the control group. Unpaired t-test, * < 0.05, ** < 0.01, n-numbers (CG/IG): 12/17.

To analyze whether categorical variables are dependent or independent of each other, the chi-square test was applied with GraphPad Prism 9.5.1. This concerns the analysis of the quantity distribution of drop-outs, relapses, sex, and recruitment site in the CG and the IG shown in **Figures 1B** and **2B** and **Supplementary Table 4.**

**Figure 3:**
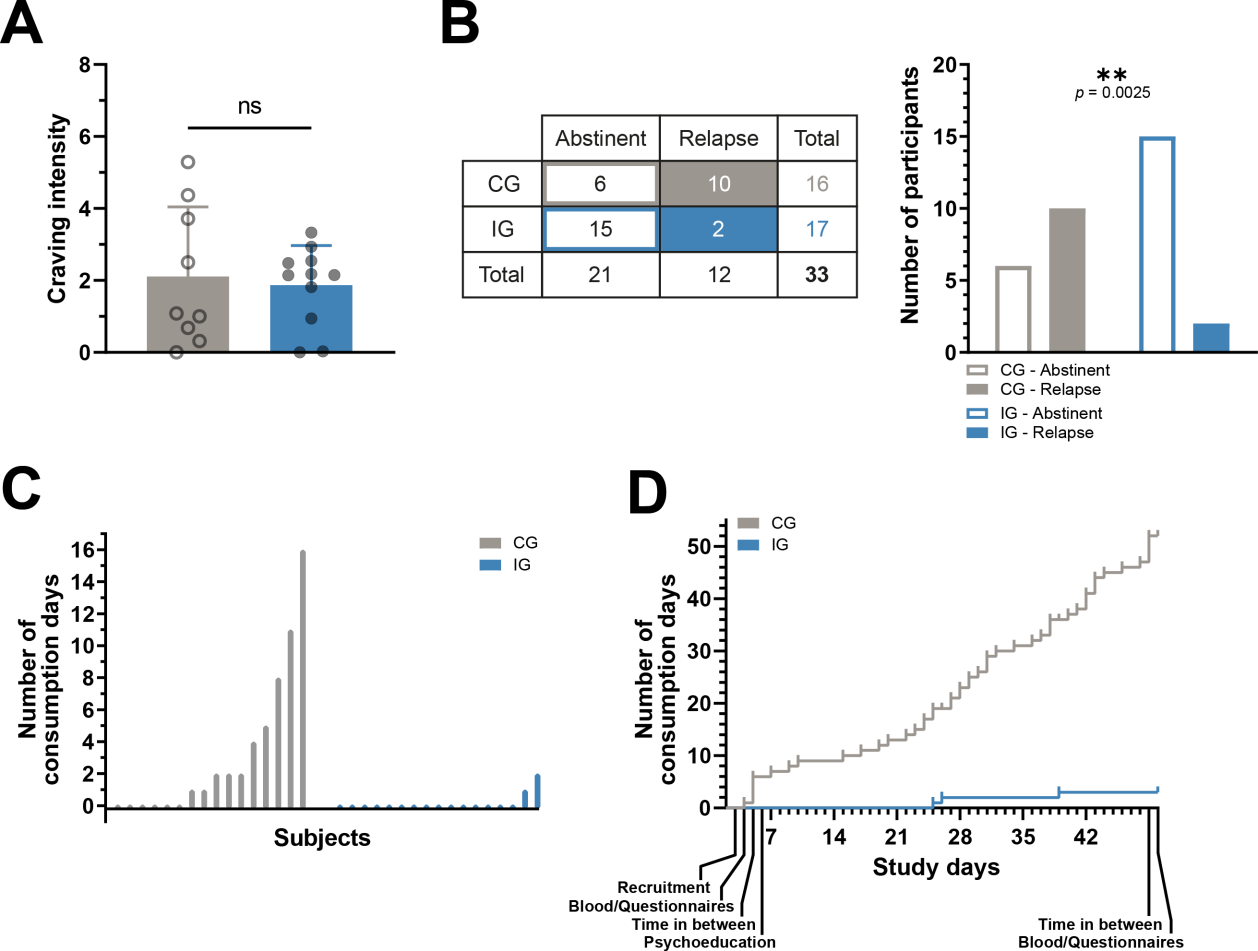
Primary outcomes. Fewer relapses and days on which alcohol was consumed in the intervention group. **(A)** The daily subjective craving for alcohol was assessed in a subset of participants, with no difference in average values between the control and intervention groups. Unpaired t-test, n.s., not significant, n-numbers (CG/IG): 9/11. **(B)** Left: Table showing the number of control and intervention subjects for whom it could be determined with certainty whether they remained abstinent or suffered at least one relapse during the study. Right: The ratio between abstinent and relapsed participants is significantly different in the control group and the intervention group. Chi-square test, **<0.01. **(C)** The number of days during the study on which alcohol was consumed by all 16 control and all 17 intervention subjects for whom it could be reliably determined whether they remained abstinent or relapsed. **(D)** The number of days of alcohol consumption accumulated over the course of the study for the control and intervention groups.

**Figure 4:**
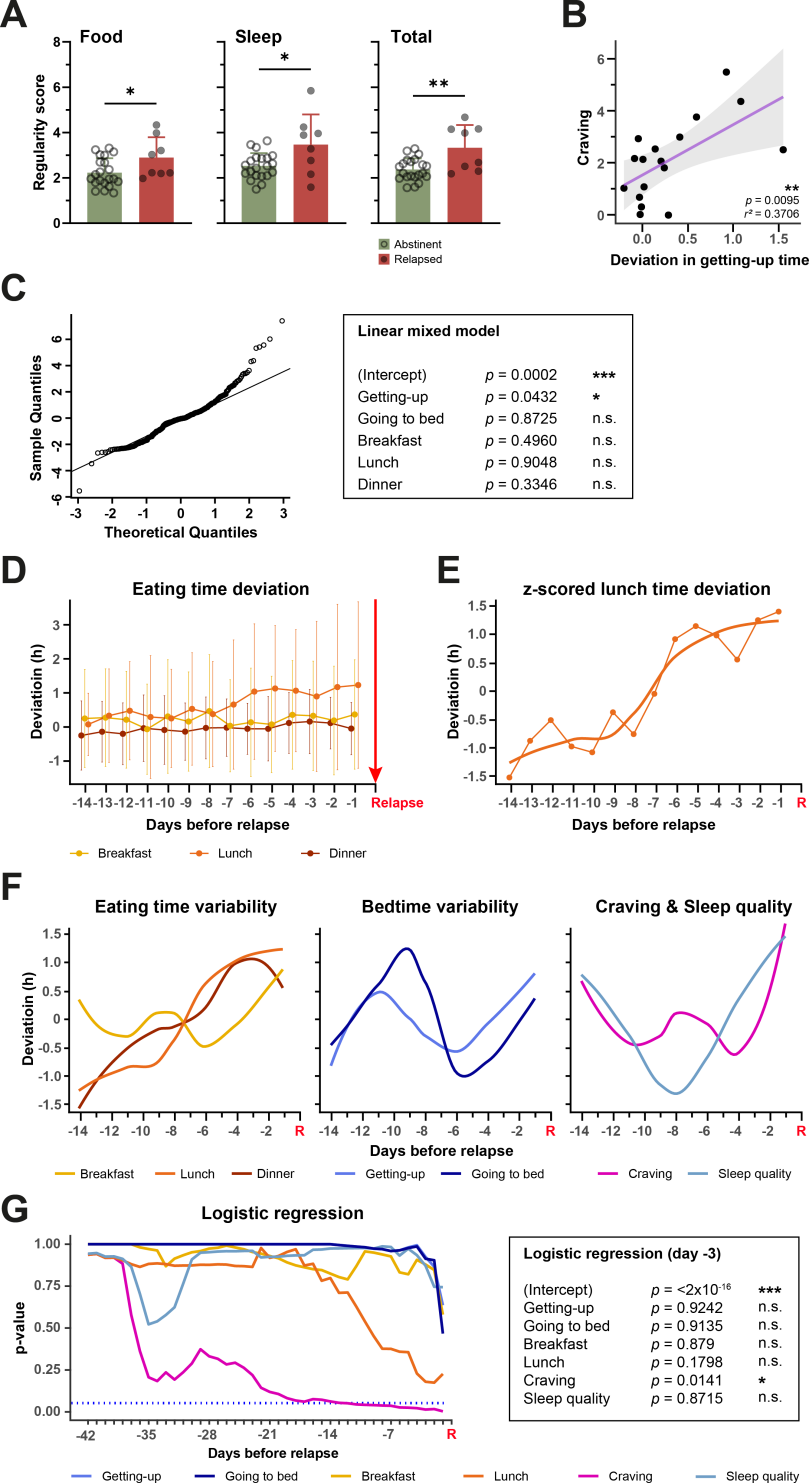
Relationships between parameters influenced by DAILY and alcohol relapses. **(A)** Abstinent participants show significantly less variability in their mealtimes and bedtimes and both together than participants who relapsed during the study. Unpaired t-test, * < 0.05, ** < 0.01, n-numbers (CG/IG): 21/8. **(B)** The average craving for alcohol of a participant is dependent on the average variability of their getting-up time. The higher the average variability of the getting-up time, the higher the average craving. Linear regression, ** < 0.01, n-number 17. **(C)** Results of a linear mixed model to investigate the influence of variability of eating times and bedtimes on each day on craving of the same day. The Quantile-Quantile (QQ) plot shows the data is normally distributed (left). **(D)** The daily variability of eating times in hours in the period of 14 days before relapse on day 0 (red arrow and marked with “Relapse”). **(E)** Z-scored deviation of lunch time with curve fit. **(F)** Curve fits of z-scored deviation of mealtimes (left), bedtimes (center) and craving and sleep quality (right). **(G)** Results of multiple logistic regressions covering decreasing numbers of days before relapse on day 0 (red R for “Relapse”) (left) with exemplary results of the logistic regression of the data from day -3 (right). * < 0.05, *** < 0.001, n.s., not significant.

To analyze whether variables of two groups differ from each other, an unpaired t-test was applied with GraphPad Prism 9.5.1. This concerns the analyses shown in **Figures 2A**, **3B**, and **4A** and **Supplementary Tables 2** and **5**.

Data presented in **Figures 4B–G** was analyzed using R version 4.2.2 and RStudio. Plots were created using the R base function *plot* and with *ggplot2* (20). To determine whether variables are related to craving, the Pearson correlation coefficient between every subject’s mean of the variable and craving score was determined. This analysis was performed with data from **Figure 4B** and **Supplementary Table 6**. For the linear mixed model shown in **Figure 4C** and **Supplementary Table 5**, subject and time were treated as random effects and all individual measures of every subject were used for fitting the model. P-values were calculated using Satterthwaite’s Approximation. It should be noted that these p-values can be misleading because of the unclear null distribution due to the unbalanced design of the model. Normality of data was confirmed with the QQ plot shown in **Figure 4D**. For the logistic regression (21) in **Figure 4G** and **Supplementary Table 8**, decreasing time windows (days -42 to -1) before each relapse of all subjects were used to obtain the respective mean for all individual parameters of that subject. If there were less than 42 days between two relapses, only the days between the two relapses were used for the analysis. The obtained averages were used to model logistic regressions for each time window with relapse as response variable.

**Figure 5:**
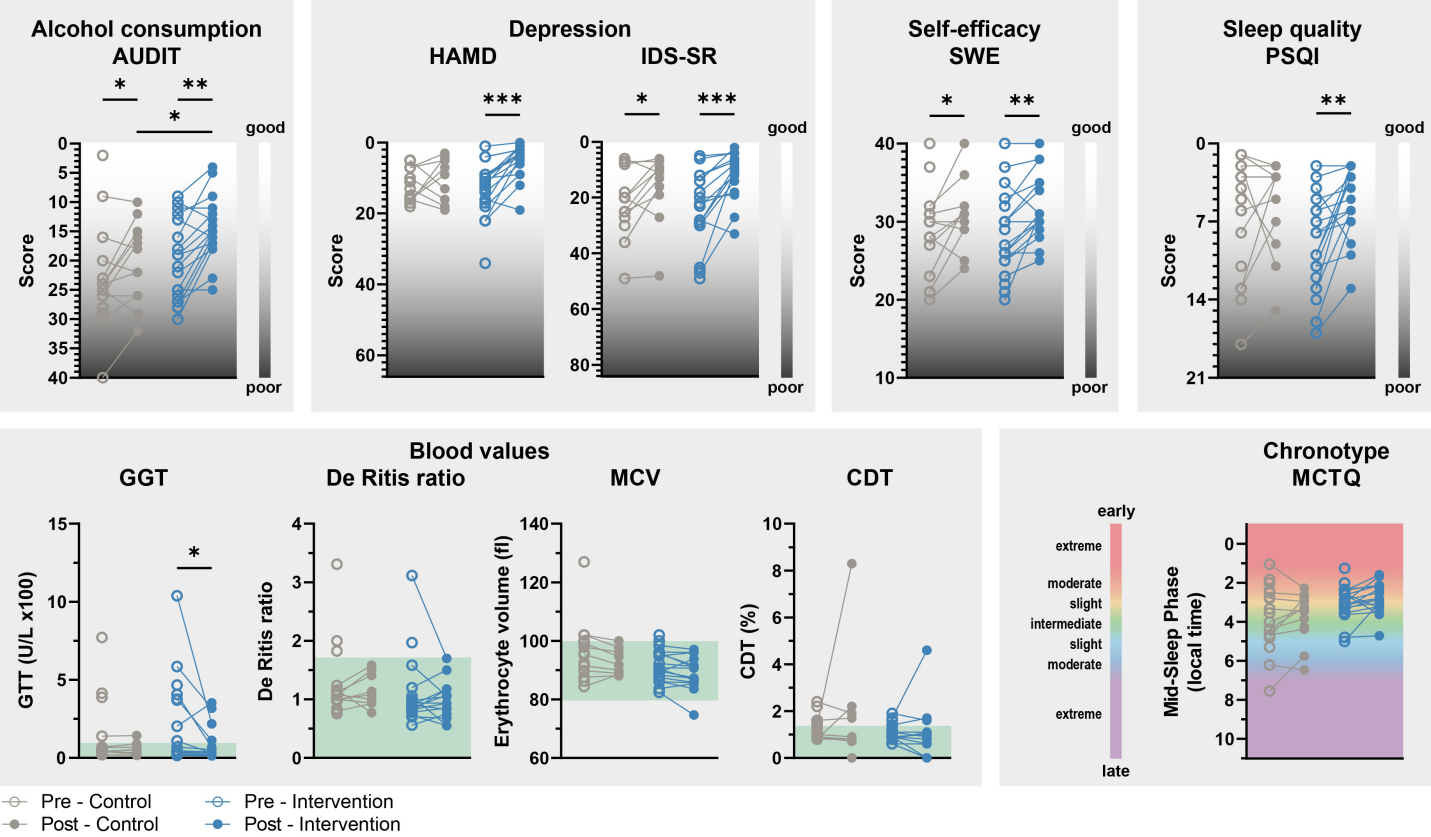
Secondary results. The improvement in AUD- and depression-related characteristics and acute liver damage over the course of the study is more pronounced in participants in the intervention group. Significant improvements are observed in various aspects of mental health: alcohol consumption (AUDIT), depressed mood (HAMD and IDS-SR), self-efficacy (SWE), and sleep quality (PSQI) in participants of the IG, but less pronounced in CG participants. Scales are either upward or downward, depending on whether low or high scores on the questionnaires mean subjectively good or poor well-being. The blood values show a significant improvement in GGT in the IG, which suggests a reduction in acute liver damage that is not observed in the CG. The green fields show the normal range of the respective blood values. The chronotype of IG and CG participants did not change during the study. 2-way repeated-measures ANOVA with Bonferroni *post hoc* test comparing CG and EG during the exploration or intervention phase and PRE and POST within groups, * p ≤ 0.05, ** p ≤ 0.01, *** p ≤ 0.001. Not all answers to the questionnaires and blood values were available from every participant. n-numbers (CG/IG): 13-18/17-20.

A mixed-effects model in GraphPad Prism 9.5.1 was used to compare group and time effects in the CG and IG and of the baseline assessment and the final appointment, as well as effects of the interaction of group and time. Additionally, Bonferroni’s multiple comparison tests were used to compare effects of time within groups (baseline assessment vs. final appointment within CG or IG) and effects between groups at either baseline assessment or final appointment (CG vs. IG at baseline assessment or final appointment). This form of analysis was applied to data in **Figure 5** and **Supplementary Table 9**. Using the same strategy, a 2-way ANOVA was used to examine the sex and group effects and their interaction in **Supplementary Table 3.**

## Results

### Participants

A total of 85 participants were screened for the pilot study, 54 of whom were recruited. Of these, 13 participants were subsequently excluded, as it was later determined that, contrary to the initial information, at least one exclusion criterion applied to them. Of the remaining 41 participants, 20 (9 women, 45%; mean age = 46.6; 9 inpatients, 45%) were randomly assigned to CG and 21 (7 women, 33.3%; mean age = 54.9; 9 inpatients, 42.9%) to IG (**Figure 1A**, **Table 1**). All of these participants had an ICD-10 diagnosis of F10.2 for alcohol dependence and 25 of them met criteria for F33.1 or F33.2 depression. There were no differences between CG and IG participants at the beginning of the study except for a significant age difference of about 8 years (**Table 1**). The evaluation of the EuropASI questionnaire also revealed no differences in the participants’ previous alcohol consumption behavior. And participants in both groups attributed equal importance to the treatment of their AUD. Nevertheless, twelve participants (6 women, 50%; 6 inpatients, 50%) dropped out of the CG during the course of the study (**Figure 1B**). This is significantly more than in the IG, in which only four participants (2 women, 50%; 3 inpatients, 75%) dropped out. These four IG participants dropped out in the first few days after the start of the study, while some of the CG participants dropped out later in the intervention phase (**Figure 1C**). Of the 12 participants who left the CG, 10 did not give a reason for leaving the study and could not be reached for comment. Two stated that they were overwhelmed in their private lives and therefore had no longer capacity to participate in the study. All four withdrawn IG subjects were no longer available at some point during the study and did not give a reason for their withdrawal. There were no significant baseline differences between completers and drop-outs, meaning that the data collected does not allow any predictions to be made about adherence to the study (**Supplementary Table 1**).

**Table 1:** Demographic and baseline data of CG and IG participants.

Characteristic	CG			IG			Difference CG - IG	
	Value	Range	SD	Value	Range	SD	p-value	Summary
sample size	20			21				
Age/sex								
Age (years)	46.6	28-65	10.39	54.86	34-72	8.979	.0095	**
Female	9			7				
Male	11			14				
Employment status								
Currently employed	12			16				
Recruitment site								
Inpatient ward	9			9				
Day-care unit	11			12				
Alcohol-related data (EuropASI)								
Age at first use	27	15-50	9.33	22	12-40	7.44	.0825	n.s.
Years of use	22	5-50	11.66	19	4-44	13.56	.4985	n.s.
Use to intoxication (years)	8	0-44	12.57	9	0-35	10.18	.7411	n.s.
Outpatient detoxications	.1	0-1	.32	.4	0-2	.58	.1351	n.s.
Inpatient detoxications	2	0-10	2.57	4.2	0-35	7.97	.2953	n.s.
Relevance of therapy (0-4)	3.3	1-4	1.02	3.5	1-4	.88	.4801	n.s.
Diagosis (ICD-10)								
F10.2	20			21				
F33.1/F33.2	11			14				
K70	8			15				
Questionnaire baseline								
AUDIT	23.67	2-40	8.260	19.05	9-30	6.962	.0698	n.s.
HAMD	11.33	5-18	4.728	13.45	1-34	7.626	.3172	n.s.
IDS-SR	22.29	6-49	11.64	25.63	5-49	13.51	.4355	n.s.
SWE	28.53	20-40	5.328	28.00	20-40	5.419	.7671	n.s.
PSQI	6.765	1-18	5.190	9.150	2-17	4.246	.1330	n.s.
MCTQ	3.730	1.0-7.5	1.681	3.025	1.3-5.0	.8641	.1097	n.s.
Blood values baseline								
GGT	138.6	21.0-771.0	208.5	216.9	16.0-1039.0	276.9	.3634	n.s.
De Ritis ratio	1.266	.76-3.31	.6386	1.097	.56-3.12	.6025	.4327	n.s.
MCV	96.79	84.4-127.0	9.871	91.65	82.4-102.0	5.621	.0676	n.s.
CDT	1.346	.80-2.40	.5142	1.094	.60-1.90	.3913	.1381	n.s.

** < 0.01; n.s., not significant.

### Improvement in the regularity of mealtimes and bedtimes

Based on the diaries, a total of 3,099 entries on daily bedtimes and 2,618 entries on eating times were collected over the course of the 5-6 weeks study period. In addition, there were 1,007 values for daily sleep quality and 548 values for daily craving for alcohol.

Using representative actograms, in which bedtime, wake-up time, and eating times are plotted over the course of 35 days, these times show more variability in CG participants than in IG participants (**Figure 2A**). A statistical comparison of the average variability scores confirms this impression, as the eating time and bedtime variability scores are significantly lower in the IG than in the CG, which is why the overall variability, taking all parameters into account, is also lower in the IG than in the CG (**Figure 2B**, **Supplementary Table 2**). A breakdown of the data by individual parameters (breakfast, lunch, dinner and getting up, going to bed) and by sex of the participants shows that the observed improvement in eating regularity is mainly due to an increase in the regularity of lunch and also tends to be driven by that of dinner (**Supplementary Table 3**). Neither getting up times nor going to bed times play a particular role in improving the regularity of sleep times (**Supplementary Table 3**). In addition, the separation shows that female and male participants appear to benefit equally from the intervention, as there is no significant effect of sex and no differences between women and men within the groups in 2-way ANOVA analyses (**Supplementary Table 3**). Between group comparison reveals, that the total score and the scores for food and lunch are significantly better for women in the IG than for women in the CG, which is not the case for men (**Supplementary Table 3**). However, it should not be concluded from this that the intervention is implemented better by women than by men, because the women in the CG had worse baseline scores than men, which is why the difference to women in the IG is more pronounced. In addition, the sample size after splitting the data is rather low, which is why final conclusions should not be drawn.

### Reduction of alcohol relapses

In those participants in whom we recorded daily craving for alcohol in the form of diary entries, there was no statistical difference in self-reported craving for alcohol between CG and IG (**Figure 3A**). Nevertheless, significantly fewer IG participants relapsed during the study than CG participants. It is not possible to say with certainty whether all 20 CG and 21 IG participants relapsed or not, as there were drop-outs who left no data and could no longer be contacted for further questions. However, of the 17 IG participants with reliable feedback regarding relapses, only two participants (<12%) had suffered relapses, while in the CG ten out of 16 participants (=62.5%) reported relapses (**Figure 3B**). In addition, the two relapsing IG participants each reported only one or two days on which they consumed alcohol, i.e. three consumption days in total. In the CG, on the other hand, some participants reported significantly more days on which alcohol was consumed. Like in the IG, five CG participants also reported only one or two days of consumption, but the others reported between four and 16 consumption days, so that there was a total of 52 days on which CG participants consumed alcohol during the study. (**Figure 3C**). Plotting the days of consumption over time shows that in the IG they occurred after 25 and 39 days respectively, while alcohol was consumed on almost every study day in the CG (**Figure 3D**). Sex and recruitment site of the participants had no significant influence on relapse (**Supplementary Table 4**). The characteristics of the relapsing subjects do not indicate that any specific group benefits particularly from the intervention (**Table 2**).

**Table 2:** Characteristics of relapsed participants.

Group	Number of relapses	Sex	Age	Recruitment site	Age at first use	Years of use	Use to intoxication (years)
**IG**	1	male	60	day-care unit	35	25	15
	2	female	42	day-care unit	25	15	12
**CG**	1	male	60	day-care unit	13	5	3
	1	female	50	day-care unit	—	—	—
	2	female	44	inpatient ward	40	4	0
	2	male	46	inpatient ward	29	16	1
	2	male	28	day-care unit	16	12	5
	4	male	44	inpatient ward	20	24	7
	5	male	56	inpatient ward	12	44	44
	8	female	32	day-care unit	27	4	—
	11	female	65	day-care unit	16	30	2
	16	male	45	day-care unit	34	11	5

---, data not available.

### Relationship between alcohol craving and relapse and day structure variability

Our results show a reduction in the variability of the daily structure on the one hand and a reduction in alcohol relapses in the IG on the other. With additional analyses, we intend to clarify whether relationships between the two variables can be identified on the basis of the data from this pilot study. Interestingly, regardless of group membership, the variability scores of eating times, bedtimes and the total variability score are on average higher in relapsing participants from whom diary data are available than in abstinent participants (**Figure 4A**, **Supplementary Table 5**).

Although there is no difference in alcohol craving between the more irregular CG and the more regular IG (**Figure 3A**), there is a significant relationship between craving and the variability of getting up times, with higher average variability associated with higher average craving (**Figure 4B**, **Supplementary Table 6**). Furthermore, there is a tendency for a correlation between sleep quality and craving. However, the average variability of this and other parameters is not significantly related to the average craving (**Supplementary Table 6**). In a linear mixed model, in which not the average of variability and craving, but the values of each individual study day are taken into account, it is confirmed that the drift in getting up time on one day can predict craving on the same day (**Figure 4C**, **Supplementary Table 7**).

In another approach, we investigated whether there was a change in the daily parameters or their variability in the days before a relapse, which could possibly have predicted the relapse. Using the example of eating times, it can be seen that the day-to-day variability of lunch and dinner times continues to increase before relapses (**Figure 4D**). This increase in variability becomes even clearer when the data is z-scored and a curve fit is placed along this data, as in the example of lunch variability (**Figure 4E**). The comparison of the curve fits of all parameters shows that the variability of lunch and dinner time in particular increases continuously over the course of 14 days until the relapse occurs (**Figure 4F**). Craving also rises sharply before a relapse and in the days before the relapse clearly exceeds the range in which it previously fluctuated. This is not the case with the variability of breakfast time, getting-up time, going-to-bed time, and sleep quality. Although an increase in these variables can also be observed before the relapse, it differs less from the fluctuations that occur on other days without a subsequent relapse. A logistic regression shows that the change in craving is a significant predictor of relapse in the entire period up to approximately 12 days before a relapse (**Figure 4G**, **Supplementary Table 8**). Although the change in lunchtime variability does not reach significance as a predictor, it comes much closer to it over a wide time window than the variability of the other parameters.

### Improvement of secondary symptoms of AUD

The evaluation of the questionnaires and blood values collected at the beginning and end of the study almost consistently shows a more efficient improvement in secondary symptoms of AUD in the IG than in the CG (**Figure 5**, **Supplementary Table 9**). Importantly, the baseline levels at the beginning of the study did not differ in CG and IG (**Table 1**). Alcohol consumption (AUDIT) in the last four weeks improved significantly in both groups, but more markedly in the IG than in the CG, leading to a significant difference between the two groups at the end of the study. Clinician-rated depressive symptoms (HAMD) improved exclusively in the IG. Self-rated depressive symptoms (IDS-SR) also showed a significant improvement in the CG, although this was even more pronounced in the IG. The same applies to subjective self-efficacy (SWE). In addition, sleep quality (PSQI) improved during the study in the IG, but not in the CG.

The GGT blood value, which is an indicator of acute liver damage, only improved significantly in the IG. There were no significant changes in the De Ritis ratio, which also indicates acute liver damage, the MCV value, which measures blood cell volume, and CDT, which reflects alcohol consumption in previous days, either in the CG or in the IG.

Consistent with our aim to optimize rather than to shift individual rhythms, chronotype (MCTQ) did not change during the study.

## Discussion

In our DAILY pilot study, we have obtained promising results showing that an intervention to reduce the variability of daily bedtimes and mealtimes leads to a significant reduction in relapse and days of alcohol consumption in AUD patients who are at the beginning of detoxification and withdrawal treatment - a phase in which the risk of relapse is particularly high (22, 23). In our view, these positive results justify the inclusion of further participants in the DAILY program and the continuation and expansion of the study.

All participants in the study were patients in our clinic’s addiction ward or day clinic and received the same basic treatment for their AUD. IG participants were additionally offered the DAILY intervention to reduce variability in their daily structure. CG participants also received an additional treatment of the same scope, which did not focus on daily structure, but on dealing with confrontation with the advertising and marketing of alcoholic beverages and other legal substances in public. Thus, the difference between CG and IG merely relates to the content discussed; the same data was collected in both groups and data collection took place in the same way.

The AUDIT questionnaire shows a significant improvement in drinking behavior in both the CG and the IG - both standard therapy and standard therapy plus DAILY are therefore effective. However, the improvement in the AUDIT score was more pronounced in the IG than in the CG and the scores of both groups differed significantly from each other at the end of the study. In our view, this result is very convincingly supported by the diary data. On the one hand, the diary entries show that despite standard treatment, over 60% of the CG participants who provided reliable data in this regard suffered at least one relapse during the study, while in the IG only under 12% of the participants relapsed. On the other hand, they show that the relapses in the IG were rather exceptions, as the two participants concerned only had one or two days on which they drank alcohol during the course of the study, whereby the two days of consumption of one participant directly followed each other and can therefore possibly be assessed as only one relapse. In the CG, on the other hand, there were numerous participants who had significantly more days of consumption, sometimes more than ten. In addition, in the CG, days of consumption occurred evenly at almost every point in the study, whereas in the IG, relapses were only recorded significantly later after more than 20 or 30 study days. This means that the relapses of the two relapsing IG participants were at least significantly delayed, and their overall consumption was limited despite their increased susceptibility.

Importantly, our analyses indicate that the low number of relapses of participants in the IG is related to the improvement of their daily structure. On the one hand, abstinent participants show on average significantly lower variability in their daily activities than relapsing participants. On the other hand, the craving for alcohol on a particular day is lower the more precisely the get-up time on that day is maintained. Secondly, relapses are preceded by a successive increase in the variability of lunch and dinner times. The variability of the times for breakfast, getting up, and going to bed and a worsening of sleep quality also increase before a relapse, but they also do so on days that are not followed by a relapse, which is why they cannot be used alone as predictors. An increase in craving also precedes a relapse. In a logistic regression, only craving can significantly predict a relapse several days in advance. However, the predictive ability of other parameters also approaches the significance threshold, especially shortly before a relapse occurs. This is particularly evident in the variability of lunch time. Taken together, craving is related to the variability of getting up time. And observing increases in craving in combination with increases in temporal variability of certain parameters and deterioration in sleep quality may be the most reliable way to predict the risk of a relapse.

In addition to the primary outcome of reducing relapses, the questionnaire data also show a more efficient improvement in depressive symptoms (both subjective and clinician-assessed) and sleep quality and an increased sense of self-efficacy through DAILY. However, no change in chronotypes was observed, which is in line with our aim to strengthen and not shift individual circadian rhythms. The results of the blood analyses, on the other hand, are less clear. There were no differences between CG and IG in the De Ritis Ratio, MCV, and CDT. Only GGT, which allows conclusions to be drawn about acute liver damage, shows a significant improvement in the IG but not in the CG. On the one hand, this can be explained by the lower alcohol consumption in the IG. However, no values from the end of the study are available from those CG participants who had particularly high GGT values at the beginning of the study, so it remains unclear whether their values might not have improved. Furthermore, other possible causes of liver damage were not investigated, therefore no reliable conclusion can be drawn about alcohol-related causes. The drop-out rate can be used as a further indication that AUD patients apparently benefit from DAILY. 60% of all recruited CG participants dropped out, some of them later in the study. In contrast, only just under 20% of participants in the IG withdrew from the study, and this tended to be at the beginning, when the intervention had not yet started or had only just begun. This suggests that, in addition to the objective improvement in their condition, the IG participants also benefited subjectively from the DAILY intervention and showed correspondingly higher compliance than CG participants.

Despite the promising results of the pilot study, the implementation to date has some limitations that need to be overcome when continuing the study.

A central hypothesis of our work is that precise adherence to a daily structure leads to an improvement in endogenous circadian rhythms, which are responsible for a reduction in relapse, days of consumption, depression, and other secondary symptoms of AUD. With the exception of behavioral rhythms, however, we have not collected any data in the present pilot study that actually prove a change in rhythms at the endogenous level. The study should therefore be expanded in the future to include further measurements, such as blood and saliva samples or temperature measurements, which will be taken at least at the beginning and end of the study over the course of 24 hours in order to be able to draw more reliable conclusions about the endogenous rhythms of the participants.

In addition, the diary data is self-reported data, some of which was entered in paper form - a method that is prone to underreporting and insufficient compliance. The implication of application-based data collection via smartphone (as done for four of the last participants of this pilot study) would be desirable for the continuation of the study. In order to further increase the objectivity of the data, the parallel use of actimetry is also being sought.

For the collection of the present data, participants were recruited from both inpatients and day patients at our clinic. Both groups differed in terms of their ability to determine their own daily structure, but both had certain guidelines from the clinical side regarding their daily structure. In addition, they often differed in terms of how they continued their treatment after completing therapy at our clinic. Many of the inpatient participants began long-term therapy in another facility after discharge from the ward or transferred to the day-care setting of our clinic, and many of the day-care participants continued their daily lives and the study at home. This results in a very heterogeneous group, of which both the CG and IG participants were at least temporarily in an environment in which they were given a certain degree of daily structure. As a result, even the CG participants showed less variability than people without an AUD diagnosis in their normal daily routine (Ref.: TTE). For the future resumption of the DAILY study, this means that a purely outpatient setting is more suitable for achieving greater differences in daily structure variability between CG and IG. In addition, the outpatient setting is more suitable from a therapeutic perspective, as patients can organize their daily structure in a self-determined manner and are likely to benefit most from supervised therapy to reduce temporal variability in their daily activities.

The relatively short duration of the DAILY intervention is also unsuitable for a continuation of the study, because even if the risk of relapse decreases over time after the start of withdrawal therapy, the first relapses can occur even after longer abstinence phases (22, 23). From a scientific perspective, it would be interesting to find out whether IG participants remain abstinent in the long term and how long it takes before a relapse occurs. And from a therapeutic perspective, the implementation of DAILY probably makes sense over a long period of time until patients are able to maintain a stable daily structure permanently, independently, and with ease.

As this is a pilot study, the sample size is too small to be able to draw reliable conclusions. This not only affects the robustness of the analyses conducted here, but also hinders analyses that would only have been possible with a larger number of participants. Our results to date indicate, for example, that activities such as getting up, breakfast, and lunch in particular are associated with craving and relapse. However, the small sample size prevented us from carrying out analyses that would identify those activities for each individual participant whose temporal variability is particularly closely associated with craving and relapse. In addition, the small sample size does not allow a robust analysis of whether certain circadian characteristics are risk factors for craving for alcohol, alcohol consumption, and relapse. We believe that strengthening circadian rhythms with all their individual characteristics, rather than adjusting the rhythms to a moderate chronotype, for example, is the best way to improve health deficits as it does not counteract genetically predetermined traits and is easier for participants to implement (19). However, other studies show that eveningness in particular, i.e., a late chronotype, is a risk factor for harmful alcohol consumption (24, 25). If this proves to be the case in a future, more comprehensive data set, it could be useful to offer daily structure plans with rather early times as a therapeutic measure for patients with late chronotypes. A sub-study would be appropriate here, with late chronotypes that become phase advanced and those that can remain late, as it is as yet uncertain whether being late is problematic in itself (e.g. for social reasons) or whether the same genetic constellation that causes a late chronotype is also responsible for increased alcohol consumption.

Despite these limitations, the data obtained so far provide reason to believe that DAILY as an adjunctive therapeutic measure can significantly increase the efficacy of standard treatments for AUD. If the results found here are confirmed with a larger sample size and after adjustment of the above-mentioned limitations, DAILY would be an easy-to-implement and effective tool for therapists to reduce relapse during and after withdrawal therapy. In particular, the analyses to identify those parameters whose temporal variability is particularly strongly related to craving and those whose increase in variability precedes relapse would be valuable for therapists and patients. First, some patients may find it easier to implement DAILY if they have to keep the variability of only some daily activities low in order to achieve success. Second, if therapists and patients observe an increase in craving and an increase in the variability of certain activities, they can counteract this in a targeted manner and thus possibly reduce the risk of an imminent relapse.

## Data Availability

The raw data supporting the conclusions of this article will be made available by the authors, without undue reservation.
