# Supplementary material for: DAILY – A personalized circadian Zeitgeber therapy as an adjunctive treatment for alcohol use disorder patients: results of a pilot trial
Source: Front Psychiatry. 2025 Feb 7;16:1477895. doi: 10.3389/fpsyt.2025.1477895 (PMC11842383; doi:10.3389/fpsyt.2025.1477895)
Supplement: Supplementary file 1 [file Table1.docx]

**Supplementary Table 1**: Statistical values of Kruskal-Wallis tests with Dunn’s multiple comparison tests; Completers vs. Drop-outs in CG and IG.

| Parameter | Variable value (mean ± SD) | | | | n-value | | | | Statistical test | p-value | Summary | Dunn’s multiple comparison test | | | |
| --- | --- | --- | --- | --- | --- | --- | --- | --- | --- | --- | --- | --- | --- | --- | --- |
|  | CG | | IG | | CG | | IG | |  |  |  | CG_C vs. CG_D | | IG_C vs. IG_D | |
|  | Completers | Drop-outs | Completers | Drop-outs | C | D | C | D |  |  |  | p-val. | Sum. | p-val. | Sum. |
| Age | 45.13 ± 12.49 | 47.58 ± 9.19 | 55.00 ± 7.96 | 54.25 ± 14.1 | 8 | 12 | 17 | 4 | Kruskal-Wallis test | .0916 | n.s. | >.9999 | n.s. | >.9999 | n.s. |
| Age at first use | 20.86 ± 7.31 | 22.70 ± 8.21 | 26.38 ± 8.04 | 31.33 ± 17.6 | 7 | 10 | 16 | 3 |  | .4186 | n.s. | >.9999 | n.s. | >.9999 | n.s. |
| Years of use | 24.71 ± 18.2 | 14.8 ± 9.04 | 23.75 ± 11.8 | 11.67 ± 7.64 | 7 | 10 | 16 | 3 |  | .1950 | n.s. | .4306 | n.s. | .2339 | n.s. |
| Use to intoxication (years) | 16.40 ± 19.5 | 4.00 ± 6.07 | 10.83 ± 10.9 | 3.33 ± 5.78 | 5 | 10 | 16 | 3 |  | .2900 | n.s. | .3733 | n.s. | .3688 | n.s. |
| Outpatient detoxications | .00 ± .00 | .20 ± .42 | .44 ± .63 | .00 ± .00 | 7 | 10 | 16 | 3 |  | .1730 | n.s. | .7021 | n.s. | .3102 | n.s. |
| Inpatient detoxications | 2.29 ± 3.55 | 1.80 ± 1.99 | 4.94 ± 8.77 | .33 ± .577 | 7 | 10 | 16 | 3 |  | .2860 | n.s. | >.9999 | n.s. | .1216 | n.s. |
| Relevance of therapy (0-4) | 3.14 ± 1.07 | 3.40 ± 1.08 | 3.50 ± .97 | 3.67 ± .58 | 7 | 10 | 16 | 3 |  | .8564 | n.s. | >.9999 | n.s. | >.9999 | n.s. |
| AUDIT | 25.00 ± 8.73 | 22.60 ± 8.17 | 19.53 ± 6.85 | 16.33 ± 8.51 | 9 | 10 | 17 | 3 |  | .3101 | n.s. | >.9999 | n.s. | >.9999 | n.s. |
| HAMD | 11.38 ± 5.04 | 11.30 ± 4.74 | 11.76 ± 5.92 | 23.00 ± 10.5 | 8 | 10 | 17 | 3 |  | .2343 | n.s. | >.9999 | n.s. | .0954 | n.s. |
| IDS-SR | 22.31 ± 11.43 | 22.44 ± 12.5 | 23.13 ± 12.3 | 39.00 ± 14.0 | 8 | 9 | 16 | 3 |  | .3794 | n.s. | >.9999 | n.s. | .2015 | n.s. |
| SWE | 27.75 ± 3.92 | 29.22 ± 6.50 | 28.71 ± 5.38 | 24.00 ± 4.36 | 8 | 9 | 17 | 3 |  | .5359 | n.s. | >.9999 | n.s. | .3410 | n.s. |
| PSQI | 4.25 ± 4.20 | 9.00 ± 5.15 | 8.82 ± 4.36 | 11.0 ± 3.61 | 8 | 9 | 17 | 3 |  | **.0449** | ***** | .0679 | n.s. | .7844 | n.s. |
| MCTQ | 3.81 ± 1.55 | 3.66 ± 1.88 | 3.16 ± .77 | 2.25 ± 1.15 | 8 | 9 | 17 | 3 |  | .3305 | n.s. | .8701 | n.s. | .4607 | n.s. |

SD = Standard deviation

CG = Control Group

IG = Intervention Group

C = Completers

D = Drop-outs

d.f. = degree of freedom

n.s. = not significant

**Supplementary Table 2**: Statistical values of unpaired t-tests; Control Group vs. Intervention Group.

| Fig. | Parameter | Variable value (mean ± SD) | | n-value | | Statistical test | d.f. | t-value | Significance | |
| --- | --- | --- | --- | --- | --- | --- | --- | --- | --- | --- |
|  |  | CG | IG | CG | IG |  |  |  | p-value | Summary |
| 2B | Food | 2.841 ± .8688 | 2.110 ± .5142 | 12 | 17 | unpaired t-test | 27 | 2.844 | **.0084** | ****** |
|  | Bedtime | 3.185 ± 1.207 | 2.502 ± .5405 |  |  |  |  | 2.069 | **.0482** | ***** |
|  | Total | 3.039 ± .8468 | 2.285 ± .4491 |  |  |  |  | 3.116 | **.0043** | ****** |
| 3A | Craving | 2.108 ± 1.936 | 1.869 ± 1.100 | 9 | 11 |  | 18 | .3477 | .7321 | n.s. |

SD = Standard deviation

CG = Control Group

IG = Intervention Group

d.f. = degree of freedom

n.s. = not significant

**Supplementary Table 3**: Statistical values of 2-way ANOVAs with subsequent Bonferroni post-hoc test analyzing sex, group, as well as interaction effects between time x sex

| Parameter | Value (mean; 95% CI) | | | | | | | | Statistical test | ANOVA results | Source of Variation | | | Bonferroni post-hoc | | | | | | | |
| --- | --- | --- | --- | --- | --- | --- | --- | --- | --- | --- | --- | --- | --- | --- | --- | --- | --- | --- | --- | --- | --- |
|  |  |  |  |  |  |  |  |  |  |  |  |  |  | Within Group  (female vs. male) | | | | Between Group  (CG vs. IG) | | | |
|  | CG | | | | IG | | | |  |  |  |  |  | CG | | IG | | female | | male | |
|  | Female | n-value | Male | n-value | Female | n-value | Male | n-value |  |  | Group | Sex | Sex x Group | p-val. | Sum. | p-val. | Sum. | p-val. | Sum. | p-val. | Sum. |
| Total | 3.285;  (2.248, 4.321) | 6 | 2.793;  (2.083, 3.503) | 6 | 2.248;  (1.628, 2.867) | 5 | 2.301;  (2.015, 2.586) | 12 | 2-way ANOVA | d.f. | 1, 25 | 1, 25 | 1, 25 | .03966 | n.s. | >.9999 | n.s. | **.0270** | ***** | .2779 | n.s. |
|  |  |  |  |  |  |  |  |  |  | F-value | 9.138 | .7515 | 1.158 |  |  |  |  |  |  |  |  |
|  |  |  |  |  |  |  |  |  |  | p-value | **.0057** | .3943 | .2921 |  |  |  |  |  |  |  |  |
|  |  |  |  |  |  |  |  |  |  | Summary | ****** | n.s. | n.s. |  |  |  |  |  |  |  |  |
| Food | 3.076;  (2.056, 4.096) | 6 | 2.736;  (1.771, 3.700) | 5 | 2.009;  (1.418, 2.600) | 5 | 2.152;  (1.807, 2.498) | 12 |  | d.f. | 1, 24 | 1, 24 | 1, 24 | .8423 | n.s. | >.9999 | n.s. | **.0337** | ***** | .2462 | n.s. |
|  |  |  |  |  |  |  |  |  |  | F-value | 8.905 | .7253 | .3908 |  |  |  |  |  |  |  |  |
|  |  |  |  |  |  |  |  |  |  | p-value | **.0064** | .7253 | .3908 |  |  |  |  |  |  |  |  |
|  |  |  |  |  |  |  |  |  |  | Summary | ****** | n.s. | n.s. |  |  |  |  |  |  |  |  |
| Sleep | 3.341;  (1.821, 4.862) | 6 | 3.029;  (1.957, 4.102) | 6 | 2.563;  (1.861, 3.265) | 5 | 2.477;  (2.126, 2.829) | 12 |  | d.f. | 1, 25 | 1, 25 | 1, 25 | >.9999 | n.s. | >.9999 | n.s. | .3336 | n.s. | .4650 | n.s. |
|  |  |  |  |  |  |  |  |  |  | F-value | 3.524 | .3154 | .1017 |  |  |  |  |  |  |  |  |
|  |  |  |  |  |  |  |  |  |  | p-value | .0722 | .7524 | .5794 |  |  |  |  |  |  |  |  |
|  |  |  |  |  |  |  |  |  |  | Summary | n.s. | n.s. | n.s. |  |  |  |  |  |  |  |  |
| Breakfast | 3.323;  (1.775, 4.871) | 6 | 2.396;  (.749, 4.043) | 6 | 1.834;  (.943, 2.725) | 5 | 2.466;  (1.751, 3.181) | 10 |  | d.f. | 1, 23 | 1, 233 | 1, 23 | .4028 | n.s. | .7083 | n.s. | .1114 | n.s. | >.9999 | n.s. |
|  |  |  |  |  |  |  |  |  |  | F-value | 2.137 | .09198 | 2.576 |  |  |  |  |  |  |  |  |
|  |  |  |  |  |  |  |  |  |  | p-value | .1574 | .7644 | .1221 |  |  |  |  |  |  |  |  |
|  |  |  |  |  |  |  |  |  |  | Summary | n.s. | n.s. | n.s. |  |  |  |  |  |  |  |  |
| Lunch | 3.485;  (2.217, 4.753) | 6 | 2.185;  (1.101, 3.268) | 5 | 1.745;  (1.284, 2.206) | 5 | 2.142;  (1.671, 2.612) | 12 |  | d.f. | 1, 24 | 1, 24 | 1, 24 | **.0346** | ***** | .7684 | n.s. | **.0045** | ****** | >.9999 | n.s. |
|  |  |  |  |  |  |  |  |  |  | F-value | 6.934 | 1.782 | 6.278 |  | | | | | | | |
|  |  |  |  |  |  |  |  |  |  | p-value | **.0146** | .1944 | **.0194** |  |  |  |  |  |  |  |  |
|  |  |  |  |  |  |  |  |  |  | Summary | ***** | n.s. | ***** |  |  |  |  |  |  |  |  |
| Dinner | 2.629;  (1.562, 3.696) | 6 | 2.759;  (2.048, 3.469) | 6 | 2.254;  (1.143, 3.366) | 5 | 1.979;  (1.448, 25.10) | 12 |  | d.f. | 1, 25 | 1, 25 | 1, 25 | >.9999 | n.s. | >.9999 | n.s. | .9547 | n.s. | .1616 | n.s. |
|  |  |  |  |  |  |  |  |  |  | F-value | 2.941 | .04660 | .3629 |  | | | | | | | |
|  |  |  |  |  |  |  |  |  |  | p-value | .0987 | .8308 | .5523 |  |  |  |  |  |  |  |  |
|  |  |  |  |  |  |  |  |  |  | Summary | n.s. | n.s. | n.s. |  |  |  |  |  |  |  |  |
| Getting up | 3.705;  (1.751, 5.659) | 6 | 3.165;  (1.937, 4.393) | 6 | 2.716;  (1.239, 4.193) | 5 | 2.672;  (2.050, 3.294) | 12 |  | d.f. | 1, 25 | 1, 25 | 1, 25 | .9365 | n.s. | >.9999 | n.s. | .4207 | n.s. | .8901 | n.s. |
|  |  |  |  |  |  |  |  |  |  | F-value | 2.206 | .3431 | .2473 |  | | | | | | | |
|  |  |  |  |  |  |  |  |  |  | p-value | .1499 | .5633 | .6233 |  |  |  |  |  |  |  |  |
|  |  |  |  |  |  |  |  |  |  | Summary | n.s. | n.s. | n.s. |  |  |  |  |  |  |  |  |
| Going to bed | 2.901;  (1.554, 4.247) | 6 | 2.809;  (1.603, 4.015) | 6 | 2.388;  (.933, 3.843) | 5 | 2.273;  (1.893, 2.653) | 12 |  | d.f. | 1, 25 | 1, 25 | 1, 25 | >.9999 | n.s. | >.9999 | n.s. | .7966 | n.s. | .5746 | n.s. |
|  |  |  |  |  |  |  |  |  |  | F-value | 1.836 | .07171 | .0008784 |  | | | | | | | |
|  |  |  |  |  |  |  |  |  |  | p-value | .1876 | .7911 | .9766 |  |  |  |  |  |  |  |  |
|  |  |  |  |  |  |  |  |  |  | Summary | n.s. | n.s. | n.s. |  |  |  |  |  |  |  |  |

CG = Control Group

IG = Intervention Group

d.f. = degree of freedom

n.s. = not significant

val. =value

Sum. = Summary

**Supplementary Table 4**: Statistical values of Chi-square tests.

| Fig. | Values (counts) | | | | | Chi-square test | | | |
| --- | --- | --- | --- | --- | --- | --- | --- | --- | --- |
|  |  |  |  |  |  | Chi-square | d.f. | p-value | Summary |
| 1B | Drop-outs |  | completed | Drop-out | total |  |  |  |  |
|  |  | CG | 8 | 12 | 20 | 7.220 | 1 | **.0072** | ****** |
|  |  | IG | 17 | 4 | 21 |  |  |  |  |
|  |  | Total | 25 | 16 | 41 |  |  |  |  |
| 3B | Relapses |  | abstinent | relapsed | total |  |  |  |  |
|  |  | CG | 6 | 10 | 16 | 9.169 | 1 | **.0025** | ****** |
|  |  | IG | 15 | 2 | 17 |  |  |  |  |
|  |  | Total | 21 | 12 | 33 |  |  |  |  |
|  | Sex |  | abstinent | relapsed | total |  |  |  |  |
|  |  | Female | 7 | 5 | 12 | .2292 | 1 | .6321 | n.s. |
|  |  | Male | 14 | 7 | 21 |  |  |  |  |
|  |  | Total | 21 | 12 | 33 |  |  |  |  |
|  | Site |  | abstinent | relapsed | total |  |  |  |  |
|  |  | Day-care unit | 13 | 8 | 21 | .07483 | 1 | .7844 | n.s. |
|  |  | Inpatient ward | 8 | 4 | 12 |  |  |  |  |
|  |  | Total | 21 | 12 | 33 |  |  |  |  |

CG = Control Group

IG = Intervention Group

d.f. = degree of freedom

n.s. = not significant

**Supplementary Table 5**: Statistical values of unpaired t-tests; Abstinent vs. Relapsed.

| Fig. | Parameter | Variable value (mean ± SD) | | n-value | | Statistical test | d.f. | t-value | Significance | |
| --- | --- | --- | --- | --- | --- | --- | --- | --- | --- | --- |
|  |  | CG | IG | CG | IG |  |  |  | p-value | Summary |
| 4A | Food | 2.229 ± .6368 | 2.896 ± .8944 | 21 | 8 | unpaired t-test | 27 | 2.254 | **.0325** | ***** |
|  | Bedtime | 2.525 ± .5658 | 3.467 ± 1.333 |  |  |  |  | 2.714 | **.0114** | ***** |
|  | Total | 2.367 ± .4934 | 3.329 ± 1.005 |  |  |  |  | 3.481 | **.0017** | ****** |

SD = Standard deviation

CG = Control Group

IG = Intervention Group

d.f. = degree of freedom

n.s. = not significant

**Supplementary Table 6**: Statistical values of linear regressions.

| Fig. | Variables | n-value | Statistical test | Slope | R² | Is the slope significantly non-zero? | | | |
| --- | --- | --- | --- | --- | --- | --- | --- | --- | --- |
|  |  |  |  |  |  | t-value | d.f. | p-value | Summary |
| 4B | Getting-up vs. Craving | 17 | Linear Regression | 1.937972 | .37058 | 2.9718 | 15 | **.0095** | ****** |
|  | Going to bed vs. Craving | 17 | Linear Regression | 1.65842 | .05878 | .935 | 14 | .3656 | n.s. |
|  | Breakfast vs. Craving |  |  | 1.039031 | .06602 | 1.0297 | 15 | .3195 | n.s. |
|  | Lunch vs. Craving |  |  | 1.6108 | .08498 | 1.1403 | 14 | .2733 | n.s. |
|  | Dinner vs. Craving |  |  | -2.163527 | .09936 | -1.286 | 15 | .2178 | n.s. |
|  | Sleep quality vs. Craving |  |  | -.501785 | .16062 | -1.856 | 18 | .07992 | n.s. |

d.f. = degree of freedom

n.s. = not significant

**Supplementary Table 7**: Statistical values of linear mixed model.

| Fig. | Parameter | Estimate | t-value | p-value | Summary |
| --- | --- | --- | --- | --- | --- |
|  |  |  |  |  |  |
| 4C | Intercept | 2.18458 | 5.066 | **.000212** | ******* |
|  | Getting-up | .15494 | 2.031 | **.043145** | ***** |
|  | Going to bed | -.02180 | -.161 | .872514 | n.s. |
|  | Breakfast | -.10369 | -.682 | .495985 | n.s. |
|  | Lunch | -.01472 | -.120 | .904815 | n.s. |
|  | Dinner | .13304 | .966 | .334501 | n.s. |

n.s. = not significant

**Supplementary Table 8**: Statistical values of logistic regressions (day -3).

| Fig. | Parameter | Estimate | Standard deviation | z-value | p-value | Summary |
| --- | --- | --- | --- | --- | --- | --- |
| 4G | Intercept | -3.22552 | .16995 | -18.98 | **<2x10^-16^** | ******* |
|  | Getting-up | .02256 | .23721 | .095 | .9242 | n.s. |
|  | Going to bed | .02577 | .23721 | .109 | .9135 | n.s. |
|  | Breakfast | .03742 | .24583 | .152 | .879 | n.s. |
|  | Lunch | -.35916 | .26778 | -1.341 | .1798 | n.s. |
|  | Craving | .59443 | .2422 | 2.454 | **.0141** | ***** |
|  | Sleep quality | -.03915 | .24196 | -.162 | .8715 | n.s. |

n.s. = not significant

**Supplementary Table 9**: Statistical values of Mixed effect models with subsequent Bonferroni post-hoc test analyzing time, group, as well as interaction effects between time x group

| Fig. | Para-meter | Value (mean; 95% CI) | | | | | | | | Statistical test | ANOVA results | Source of Variation | | | Bonferroni post-hoc | | | | | | | |
| --- | --- | --- | --- | --- | --- | --- | --- | --- | --- | --- | --- | --- | --- | --- | --- | --- | --- | --- | --- | --- | --- | --- |
|  |  |  |  |  |  |  |  |  |  |  |  |  |  |  | Within Group  (beginning vs. end) | | | | Between Group  (CG vs. IG) | | | |
|  |  | CG | | | | IG | | | |  |  |  |  |  | CG | | IG | | beginning | | end | |
|  |  | Beginning | n-value | End | n-value | Beginning | n-value | End | n-value |  |  | Group | Time | Time x Group | p-val. | Sum. | p-val. | Sum. | p-val. | Sum. | p-val. | Sum. |
| 5 | AUDIT | 23.67;  (19.56, 27.77) | 18 | 20.42;  (16.04, 24.79) | 12 | 19.05;  (15.79, 22.31) | 20 | 14.59;  (11.78, 17.39) | 17 | Mixed-effects model | d.f. | 1, 36 | 1, 27 | 1, 27 | **.0488** | ***** | **.0011** | ****** | .1027 | n.s. | **.0348** | ***** |
|  |  |  |  |  |  |  |  |  |  |  | F-value | 5.723 | 19.04 | .5341 |  |  |  |  |  |  |  |  |
|  |  |  |  |  |  |  |  |  |  |  | p-value | **.0221** | **.0002** | .4712 |  |  |  |  |  |  |  |  |
|  |  |  |  |  |  |  |  |  |  |  | Summary | ***** | ******* | n.s. |  |  |  |  |  |  |  |  |
|  | HAMD | 11.33;  (8.98, 13.68) | 18 | 10.44;  (5.67, 15.22) | 9 | 13.45;  (9.88, 17.02) | 20 | 5.13;  (2.47, 7.78) | 16 |  | d.f. | 1, 36 | 1, 23 | 1, 23 | >.9999 | n.s. | **.0001** | ******* | .5842 | n.s. | .1634 | n.s. |
|  |  |  |  |  |  |  |  |  |  |  | F-value | .3674 | 11.81 | 6.467 |  |  |  |  |  |  |  |  |
|  |  |  |  |  |  |  |  |  |  |  | p-value | .5482 | **.0022** | **.0182** |  |  |  |  |  |  |  |  |
|  |  |  |  |  |  |  |  |  |  |  | Summary | n.s. | ****** | ***** |  |  |  |  |  |  |  |  |
|  | IDS-SR | 22.29;  (16.31, 28.28) | 17 | 15.67;  (8.12, 23.22) | 12 | 25.63;  (19.12, 32.14) | 19 | 12.19;  (7.68, 16.70) | 16 |  | d.f. | 1, 34 | 1, 26 | 1, 26 | **.0308** | ***** | **.0001** | ******* | .7914 | n.s. | >.9999 | n.s. |
|  |  |  |  |  |  |  |  |  |  |  | F-value | .08129 | 26.41 | 1.539 |  |  |  |  |  |  |  |  |
|  |  |  |  |  |  |  |  |  |  |  | p-value | .7773 | **<.0001** | .2259 |  |  |  |  |  |  |  |  |
|  |  |  |  |  |  |  |  |  |  |  | Summary | n.s. | ******** | n.s. |  |  |  |  |  |  |  |  |
|  | SWE | 28.53;  (25.79, 31.27) | 17 | 30.75;  (28.03, 33.47) | 12 | 28.00;  (25.46, 30.54) | 20 | 31.53;  (29.20, 33.86) | 17 |  | d.f. | 1, 35 | 1, 27 | 1, 27 | **.0155** | ***** | **.0036** | ****** | >.9999 | n.s. | >.9999 | n.s. |
|  |  |  |  |  |  |  |  |  |  |  | F-value | .09510 | 19.68 | .002245 |  |  |  |  |  |  |  |  |
|  |  |  |  |  |  |  |  |  |  |  | p-value | .7596 | **.0001** | .9626 |  |  |  |  |  |  |  |  |
|  |  |  |  |  |  |  |  |  |  |  | Summary | n.s. | ******* | n.s. |  |  |  |  |  |  |  |  |
|  | PSQI | 6.765;  (4.096, 9.433) | 17 | 5.500;  (2.852, 8.148) | 12 | 9.150;  (7.163, 11.14) | 20 | 5.294;  (3.650, 6.938) | 17 |  | d.f. | 1, 35 | 1, 27 | 1, 27 | .8562 | n.s. | **.0034** | ****** | .1887 | n.s. | >.9999 | n.s. |
|  |  |  |  |  |  |  |  |  |  |  | F-value | .6867 | 8.275 | 2.741 |  | | | | | | | |
|  |  |  |  |  |  |  |  |  |  |  | p-value | .4129 | **.0078** | .1094 |  |  |  |  |  |  |  |  |
|  |  |  |  |  |  |  |  |  |  |  | Summary | n.s. | ****** | n.s. |  |  |  |  |  |  |  |  |
|  | GGT | 138.6;  (27.51, 249.7) | 16 | 55.67;  (23.84, 87.50) | 9 | 216.9;  (79.25, 354.6) | 18 | 85.88;  (27.10, 144.7) | 16 |  | d.f. | 1, 32 | 1, 23 | 1, 23 | >.9999 | n.s. | **.0434** | ***** | .5318 | n.s. | >.9999 | n.s. |
|  |  |  |  |  |  |  |  |  |  |  | F-value | .3455 | 3.942 | 1.090 |  | | | | | | | |
|  |  |  |  |  |  |  |  |  |  |  | p-value | .5608 | .0591 | .3072 |  |  |  |  |  |  |  |  |
|  |  |  |  |  |  |  |  |  |  |  | Summary | n.s. | n.s. | n.s. |  |  |  |  |  |  |  |  |
|  | De Ritis ratio | 1.266;  (.926, 1.606) | 16 | 1.150;  (.933, 1.368) | 9 | 1.097;  (.797, 1.397) | 18 | .987;  (.810, 1.164) | 15 |  | d.f. | 1, 32 | 1, 22 | 1, 22 | >.9999 | n.s. | .8382 | n.s. | .7502 | n.s. | .3318 | n.s. |
|  |  |  |  |  |  |  |  |  |  |  | F-value | 1.638 | .07168 | .5671 |  | | | | | | | |
|  |  |  |  |  |  |  |  |  |  |  | p-value | .2098 | .7914 | .4594 |  |  |  |  |  |  |  |  |
|  |  |  |  |  |  |  |  |  |  |  | Summary | n.s. | n.s. | n.s. |  |  |  |  |  |  |  |  |
|  | MCV | 96.79;  (91.53, 102.1) | 16 | 92.89;  (89.49, 96.28) | 9 | 91.65;  (88.86, 94.45) | 18 | 89.00;  (85.97, 92.03) | 16 |  | d.f. | 1, 32 | 1, 23 | 1, 23 | .1624 | n.s. | .0734 | n.s. | .1194 | n.s. | .1494 | n.s. |
|  |  |  |  |  |  |  |  |  |  |  | F-value | 3.662 | 7.793 | .01458 |  | | | | | | | |
|  |  |  |  |  |  |  |  |  |  |  | p-value | .0646 | **.0104** | .9050 |  |  |  |  |  |  |  |  |
|  |  |  |  |  |  |  |  |  |  |  | Summary | n.s. | ***** | n.s. |  |  |  |  |  |  |  |  |
|  | CDT | 1.346;  (1.035, 1.657) | 13 | 1.922;  (.009, 3.835) | 9 | 1.094;  (.893, 1.295) | 17 | 1.077;  (.355, 1.799) | 13 |  | d.f. | 1, 29 | 1, 19 | 1, 19 | .4468 | n.s. | >.9999 | n.s. | >.9999 | n.s. | .2246 | n.s. |
|  |  |  |  |  |  |  |  |  |  |  | F-value | 2.132 | .9109 | .9484 |  |  |  |  |  |  |  |  |
|  |  |  |  |  |  |  |  |  |  |  | p-value | .1550 | .3519 | .3424 |  |  |  |  |  |  |  |  |
|  |  |  |  |  |  |  |  |  |  |  | Summary | n.s. | n.s. | n.s. |  |  |  |  |  |  |  |  |
|  | MCTQ | 3.730;  (2.866, 4.595) | 17 | 3.774;  (2.970, 4.577) | 12 | 3.025;  (2.621, 3.429) | 20 | 2.783;  (2.382, 3.185) | 17 |  | d.f. | 1, 36 | 1, 26 | 1, 26 | .4376 | n.s. | .1766 | n.s. | .1286 | n.s. | .1286 | n.s. |
|  |  |  |  |  |  |  |  |  |  |  | F-value | 4.145 | 4.400 | .02238 |  |  |  |  |  |  |  |  |
|  |  |  |  |  |  |  |  |  |  |  | p-value | **.0492** | **.0458** | .8822 |  |  |  |  |  |  |  |  |
|  |  |  |  |  |  |  |  |  |  |  | Summary | ***** | ****** | n.s. |  |  |  |  |  |  |  |  |

CG = Control Group n.s. = not significant

IG = Intervention Group val. =value

d.f. = degree of freedom Sum. = Summary
